# Effects of fenofibrate on lipid profiles, cholesterol ester transfer activity, and in-stent intimal hyperplasia in patients after elective coronary stenting

**DOI:** 10.1186/1476-511X-9-122

**Published:** 2010-10-25

**Authors:** Tetsuro Miyazaki, Kazunori Shimada, Katsumi Miyauchi, Atsumi Kume, Kosei Tanimoto, Takashi Kiyanagi, Katsuhiko Sumiyoshi, Makoto Hiki, Hiroshi Mokuno, Shinya Okazaki, Hitoshi Sato, Takeshi Kurata, Hiroyuki Daida

**Affiliations:** 1 Department of Cardiovascular Medicine, Juntendo University School of Medicine, Tokyo, Japan

## Abstract

**Background:**

The association between modulation of detailed lipoprotein profiles and cholesterol ester transfer (CET) activity by peroxisome proliferator-activated receptor (PPAR)-a agonists in patients with coronary artery disease remains unclear. We assessed lipid profiles, plasma CET activity, and in-stent intimal hyperplasia after fenofibrate treatment in patients who underwent elective coronary stenting.

**Methods:**

Forty-three consecutive patients who underwent elective coronary stenting were randomized to the fenofibrate group (300 mg/day for 25 weeks, n = 22) or the control group (n = 21). At baseline and follow up, CET activity and lipoprotein profiles were measured, and quantitative coronary angiography was performed.

**Results:**

In the fenofibrate group, the levels of large very low-density lipoprotein cholesterol, and small low-density lipoprotein (LDL) cholesterol decreased and those of small high-density lipoprotein (HDL) cholesterol increased. Besides, CET activity decreased independent of the effect of fenofibrate on total and LDL cholesterol. The reduction of CET activity significantly correlated with the increase in LDL particle size (*r *= 0.47, *P *= 0.03) and the decrease of triglycerides in large HDL subclasses (r = 0.48, *P *= 0.03). Although there were no significant differences in restenosis parameters between the two groups, low CET activity significantly correlated with the inhibition of neointimal hyperplasia (*r *= 0.56, *P *= 0.01).

**Conclusions:**

Fenofibrate inhibited CET activity and thereby improved atherogenic lipoprotein profiles, and reduced intimal hyperplasia after coronary stenting.

## Background

Fibrates, which act as peroxisome proliferator-activated receptor (PPAR)-a agonists, are widely used in the treatment of atherogenic dyslipidemia. These drugs reduce triglycerides, raise high-density lipoprotein (HDL) cholesterol, and improve the small, dense low-density lipoprotein (LDL) phenotype [1]. Several clinical trials have demonstrated the therapeutic efficacy of fibrates in reducing cardiovascular events in patients with dyslipidemia, which predominates in patients with type 2 diabetes and metabolic syndrome [2-4].

Cholesterol ester transfer protein (CETP) plays an important role in lipoprotein metabolism, including transfer of cholesteryl ester and triglycerides between HDL and apolipoprotein B (apoB)-containing lipoproteins [5]. CETP inhibition leads to increased levels of HDL cholesterol, which contributes to prevent the initiation and progression of atherosclerosis. Indeed, CETP inhibitors have been investigated for clinical use [6,7], although controversy has also arisen [8]. CETP inhibition also results in an increase of anti-inflammatory and anti-oxidative properties of HDL conferred by apoA-1, paraoxonase 1 and platelet activating factor-acetylhydrolase [9-11]. These findings suggest CETP inhibition could be the target of treatment to prevent atherosclerotic diseases. Several studies reported that fibrates increased lipoprotein lipase activity and decreased plasma cholesterol ester transfer (CET) activity in subjects with hypertriglyceridemia and in human CETP transgenic mice [12-15]. However, the association between modulation of lipoproteins and CET activity by fenofibrate regarding the initiation and progression of atherosclerosis remains unclear.

Nowadays, in-stent restenosis remains a critical problem despite the use of newly developed drug-eluting stents [16]. Until recently, several trials using fibrates have been conducted [2-4]; however, few studies have reported a reduction of the occurrence of in-stent restenosis [17]. We have demonstrated that fenofibrate ameliorates early inflammatory responses, resulting in reduced neointimal hyperplasia after coronary stenting in a porcine model [18]. PPAR-a inhibits the expression of proinflammatory genes in a ligand-dependent manner [19,20]. In animal models, PPAR-a also inhibits intimal hyperplasia by inhibiting vascular cell recruitment and smooth muscle cell proliferation [21,22]. These results indicated the potential efficacy of fenofibrate in preventing in-stent intimal hyperplasia. In this study, we assessed the lipoprotein profile, CET activity, and in-stent intimal hyperplasia before and after fenofibrate treatment in patients who underwent elective coronary stenting.

## Methods

### Subjects

The subjects were 43 prospectively enrolled patients who underwent elective percutaneous coronary stenting at Juntendo University Hospital. Patients were randomized to the fenofibrate group (300 mg/day for 25 weeks, n = 22) or the control group (n = 21) after diagnostic coronary angiography. Randomization was undertaken to match the two groups for age, gender, body mass index (BMI), baseline lipid profiles, and presence of diabetes mellitus. All patients were instructed to follow the American Heart Association Step II diet. Fenofibrate treatment was initiated a week before coronary stenting. Before and after treatment, lipoprotein profiles and CET activity were assessed. Repeated coronary angiography was performed at baseline, just after coronary stenting and 24 weeks after coronary stenting (25-week treatment). We excluded patients with acute coronary syndrome, ongoing congestive heart failure, liver dysfunction and/or renal dysfunction. The study protocol was approved by the ethical committee of our hospital and all the patients gave their informed consent in writing.

### Blood sampling and biochemical analyses

Plasma samples were obtained immediately before coronary angiography after overnight fasting, both at baseline and after treatment. Plasma levels of total cholesterol, triglycerides, HDL cholesterol, HbA1c, and high-sensitivity C-reactive protein (hs-CRP) were measured by standard methods; LDL cholesterol levels were calculated by Friedewald's formula as previously described [23]. CET activity was measured using commercially available CETP activity assay kits (BioVision Inc, Mountain view, CA). Briefly, the CETP activity assay kit uses a donor molecule that contains a fluorescent self-quenched neutral lipid that is transferred to an acceptor molecule in the presence of CETP. CETP-mediated transfer of the fluorescent neutral lipid to the acceptor molecule results in an increased fluorescence. The value for HbA1c (%) is estimated as an NGSP equivalent value (%) calculated by the formula HbA1c (%) = HbA1c (JDS) (%) + 0.4%, considering the relational expression of HbA1c (JDS) (%) measured by the previous Japanese standard substance and measurement methods and HbA1c (NGSP) [24].

### Analysis of lipoprotein profiles

Cholesterol and triglyceride profiles in plasma lipoproteins were analyzed using the LipoSEARCH system, which is a dual-detection high performance liquid chromatography (HPLC) system with two tandem-connected TSKgel LipopropakXL columns (300 × 7.8-mm; Tosoh, Japan), at Skylight Biotech Inc. (Akita, Japan), as we and others have previously described [25,26]. We obtained the cholesterol and triglyceride levels in 20 lipoprotein subclasses according to lipoprotein particle diameter. To simplify the data analysis, we grouped some of the subclasses to yield the following 13 subclass categories: chylomicron (peak 1: > 90 nm and 2: 75 nm), large very low-density lipoprotein (VLDL) (peak 3: 64 nm, 4: 53.6 and 5: 44.5 nm), medium VLDL (peak 6: 36.8 nm), small VLDL (peak 7: 31.3 nm), large LDL (peak 8: 28.6 nm), medium LDL (peak 9: 25.5 nm), small LDL (peak 10: 23.0 nm), very small LDL (peak 11: 20.7 nm, 12: 18.6 nm and 13: 16.7 nm), very large HDL (peak 14: 15.0 nm and 15: 13.5 nm), large HDL (peak 16: 12.1 nm), medium HDL (peak 17: 10.9 nm), small HDL (peak 18: 9.8 nm), and very small HDL (peak 19: 8.8 nm and 20: 7.6 nm). The average particle diameter (nm) of LDL was obtained from the LDL peak time by HPLC.

### Angiographic methods

Coronary angiography and bare metal implantation were performed according to standard methods [27]. After intracoronary injection of isosorbide dinitrate, angiograms were obtained in 2 or more views. The technical aspects of the procedure, including the choice of bare stent and balloon, duration of inflation and pressure, were determined by each operator. Cardiac imaging system (Cardiovascular Angiography Analysis System, CAAS, Limburg, Netherlands) was used for the quantitative coronary angiography (QCA) analysis. A technician without any knowledge of the study results performed all the QCA analyses. The absolute values for the mean reference diameter and minimal luminal diameter (MLD) were determined. Percent diameter stenosis (MLD/reference), late lumen loss (MLD post minus MLD at follow-up), and loss index (late lumen loss/acute gain) were calculated. We obtained data from 19 patients in each group.

### Statistical analysis

The primary objective of the study was to assess the effect of fenofibrate on in-stent intimal hyperplasia, including minimal luminal diameter, diameter stenosis, and late lumen loss at the follow-up angiography. The secondary objectives were the effects on angiographic stenosis defined as a diameter stenosis ≥ 50% at the follow-up angiography as determined by QCA analysis; CET activity; and changes in lipid profiles defined by HPLC analysis. Data are presented as the mean ± SD. Statistical differences between the two groups were analyzed by unpaired Student's *t*-test and chi-square test. Statistical differences between baseline and post-treatment values were analyzed by paired Student's *t*-test. Correlation between two parameters was determined by simple linear regression analysis. The statistical analysis was performed using StatView software (Version 5.0 for Windows, SAS Institute, Cary, NC). The level of statistical significance was set at *P *< 0.05.

## Results

### Patient characteristics at baseline and after treatment

The characteristics of the patients at baseline and follow up are shown in Table 1. There were no significant differences in age, gender, BMI, lipid profiles, HbA1c, hs-CRP, CET activity, prevalence of diabetes mellitus and hypertension, and smoking history between the two groups at baseline. Besides, there were no significant differences between the two groups in the number of patients taking other medications, such as b-blockers (52 vs. 41%), calcium channel blockers (38 vs. 59%), angiotensin II receptor blockers (19 vs. 9%), angiotensin converting enzyme inhibitors (29 vs. 18%), statins (48 vs. 32%) or oral hypoglycemic drugs (19 vs. 18%) throughout the study period. Total and LDL cholesterol levels decreased by 9.5% (*P *= 0.01) and 12.5% (*P *= 0.01) in the control group and by 9.5% (*P *= 0.02) and 13.5% (*P *= 0.02) in the fenofibrate group. There were no significant differences in total and LDL cholesterol levels between the two groups at follow up. Fenofibrate treatment resulted in a decrease of triglyceride levels by 32.1% (*P *< 0.0001) and an increase of HDL cholesterol levels by 8.7% (*P *= 0.03) whereas no change in triglyceride or HDL cholesterol levels occurred in the control group. There were no significant changes in BMI, HbA1c or hs-CRP in either group. CET activity decreased by 30.5% in the fenofibrate group (*P *< 0.05) but it remained unchanged in the control group.

**Table 1:** Characteristics of the study population at baseline and at follow up (25 weeks of treatment)

	Baseline			Follow up		
		
	Control (n = 21)	Fenofibrate (n = 22)	*P*	Control (n = 21)	Fenofibrate (n = 22)	*P*
Age (years)	64 ± 8	67 ± 7	0.33	-	-	-
Male gender (%)	16 (76)	18 (82)	0.65	-	-	-
Diabetes mellitus (%)	6 (29)	6 (27)	0.92	-	-	-
Hypertension (%)	11 (52)	11 (50)	0.88	-	-	-
Current smoker (%)	5 (24)	6 (27)	0.80	-	-	-
Body mass index (kg/m^2^)	24.5 ± 2.5	23.7 ± 1.9	0.27	24.3 ± 2.4	23.9 ± 2.4	0.65
Total cholesterol (mg/dL)	199 ± 33	189 ± 31	0.33	180 ± 38*	171 ± 31*	0.44
Triglycerides (mg/dL)	110 ± 48	106 ± 39	0.75	102 ± 41	72 ± 37†	0.02
HDL cholesterol (mg/dL)	45 ± 11	46 ± 10	0.84	46 ± 11	50 ± 12*	0.34
LDL cholesterol (mg/dL)	110 ± 29	102 ± 24	0.32	96 ± 33*	88 ± 27*	0.40
HbA1c (%) hs-CRP (mg/L)	6.1 ± 1.4 1.33 ± 1.26	6.3 ± 1.6 1.93 ± 2.03	0.54 0.41	6.0 ± 1.3 1.63 ± 2.19	6.2 ± 1.7 1.75 ± 3.56	0.66 0.93
CET activity (pmol/μL/h)	88.4 ± 40.7	82.6 ± 50.4	0.68	94.4 ± 59.4	57.4 ± 35.1*	0.02

Data are presented as the mean ± SD. LDL = Low-density lipoprotein, HDL = High-density lipoprotein, hs-CRP = high sensitivity C-reactive protein, CET = cholesterol ester transfer. Statistical differences between the control group and the fenofibrate group were analyzed by unpaired Student's *t*-test. Statistical differences between values at baseline and at follow up were analyzed by paired Student's *t*-test. * *P *< 0.05 and † *P *< 0.01 Baseline vs. Follow up.

### Effect of fenofibrate on lipoprotein subclass profiles

Table 2 shows the changes in lipoprotein profiles analyzed by HPLC after treatment. Fenofibrate significantly decreased cholesterol levels in the chylomicron, large and medium VLDL subclasses, whereas the control group showed a significant decrease of cholesterol levels in the medium and small VLDL subclasses. Fenofibrate also significantly decreased cholesterol levels in the medium, small and very small LDL subclasses, however, there were no significant changes of cholesterol levels in any LDL subclass in the control group. In addition, fenofibrate significantly increased cholesterol levels in the medium, small and very small HDL subclasses and significantly decreased very large HDL cholesterol levels. On the contrary, the control group showed a significant increase of cholesterol levels in the very large and large HDL subclasses. Fenofibrate significantly reduced triglyceride levels in all lipoprotein subclasses except in the small and very small HDL subclasses. At follow up, no significant changes were noted in triglyceride levels in any lipoprotein in the control group.

**Table 2:** Effect of fenofibrate on lipoprotein subclass profiles analyzed by HPLC in patients with CAD

	Baseline			Follow up		
		
	Control (n = 21)	Fenofibrate (n = 22)	*P*	Control (n = 21)	Fenofibrate (n = 22)	*P*
**Cholesterol**						
Chylomicron-C (mg/dL)	0.9 ± 0.6	0.8 ± 0.6	0.54	0.7 ± 0.6	0.5 ± 0.4†	0.20
Large VLDL-C (mg/dL)	20.7 ± 6.7	20.5 ± 7.2	0.93	17.8 ± 6.3	16.1 ± 6.4†	0.41
Medium VLDL-C (mg/dL)	16.0 ± 5.0	13.8 ± 3.8	0.11	13.3 ± 4.9*	11.9 ± 3.3*	0.28
Small VLDL-C (mg/dL)	9.4 ± 2.6	8.5 ± 2.2	0.23	8.1 ± 2.4*	8.3 ± 2.2	0.72
						
Large LDL-C (mg/dL)	20.5 ± 4.7	19.8 ± 3.8	0.60	18.4 ± 5.3	19.1 ± 4.6	0.68
Medium LDL-C (mg/dL)	27.2 ± 6.2	26.9 ± 6.0	0.91	25.0 ± 8.2	23.4 ± 6.0*	0.47
Small LDL-C (mg/dL)	19.5 ± 5.0	19.7 ± 5.5	0.88	17.9 ± 6.9	15.9 ± 4.4†	0.27
Very small LDL-C (mg/dL)	14.3 ± 4.1	15.2 ± 4.6	0.48	13.2 ± 5.4	12.2 ± 3.2†	0.49
						
Very large HDL-C (mg/dL)	4.1 ± 1.2	4.7 ± 1.5	0.14	4.6 ± 1.8*	3.9 ± 1.5*	0.18
Large HDL-C (mg/dL)	7.4 ± 3.9	8.5 ± 4.1	0.37	8.8 ± 4.6*	7.1 ± 4.2	0.21
Medium HDL-C (mg/dL)	12.3 ± 3.4	12.0 ± 3.3	0.80	12.4 ± 3.4	15.4 ± 4.4†	0.02
Small HDL-C (mg/dL)	9.8 ± 2.4	9.3 ± 2.4	0.51	9.8 ± 2.1	11.9 ± 2.8†	0.009
Very small HDL-C (mg/dL)	7.0 ± 1.1	7.3 ± 1.8	0.46	6.9 ± 1.2	8.8 ± 1.8†	< 0.001
						
**Triglycerides**						
Chylomicron-TG (mg/dL)	1.9 ± 1.4	1.7 ± 1.5	0.68	1.7 ± 2.1	0.9 ± 1.0†	0.14
Large VLDL-TG (mg/dL)	22.3 ± 4.4	26.2 ± 11.5	0.15	23.7 ± 12.4	15.1 ± 9.5†	0.01
Medium VLDL-TG (mg/dL)	9.7 ± 2.3	11.0 ± 4.6	0.25	10.1 ± 4.0	7.4 ± 3.7†	0.03
Small VLDL-TG (mg/dL)	4.6 ± 1.0	5.1 ± 2.0	0.30	4.9 ± 1.7	3.8 ± 1.7†	0.05
						
Large LDL-TG (mg/dL)	6.3 ± 1.5	6.6 ± 2.0	0.54	6.3 ± 2.0	5.7 ± 2.1*	0.37
Medium LDL-TG (mg/dL)	7.2 ± 1.7	7.6 ± 1.8	0.42	6.9 ± 2.1	6.8 ± 2.2*	0.86
Small LDL-TG (mg/dL)	5.4 ± 1.3	6.1 ± 1.7	0.13	5.1 ± 1.7	5.1 ± 1.6†	0.99
Very small LDL-TG (mg/dL)	5.0 ± 1.2	5.9 ± 1.9	0.09	4.8 ± 1.7	4.6 ± 1.6†	0.68
						
Very large HDL-TG (mg/dL)	1.8 ± 0.4	1.8 ± 0.4	0.90	1.9 ± 0.6	1.2 ± 0.5†	< 0.001
Large HDL-TG (mg/dL)	2.6 ± 1.1	2.9 ± 1.2	0.40	3.3 ± 1.5	1.5 ± 1.0†	< 0.001
Medium HDL-TG (mg/dL)	4.2 ± 1.3	4.3 ± 1.2	0.77	4.5 ± 1.3	3.5 ± 1.5*	0.03
Small HDL-TG (mg/dL)	3.5 ± 1.1	3.7 ± 0.9	0.56	3.6 ± 0.9	3.5 ± 1.5	0.89
Very small HDL-TG (mg/dL)	3.2 ± 0.7	3.6 ± 0.8	0.07	3.2 ± 0.7	3.2 ± 1.2	0.95

Data are presented as the mean ± SD. CAD = coronary artery disease, VLDL = Very low-density lipoprotein, LDL = Low-density lipoprotein, HDL = High-density lipoprotein, -C = -cholesterol, -TG = -triglycerides, Statistical differences between the control group and the fenofibrate group were analyzed by unpaired Student's *t*-test. Statistical differences between values at baseline and at follow up were analyzed by paired Student's *t*-test. * *P *< 0.05 and † *P *< 0.01 Baseline vs. Follow up.

### The LDL particle size increases as CET activity is reduced by fenofibrate

**Figure 1:**
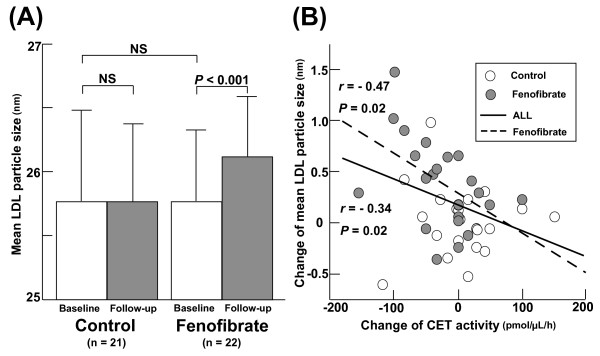
**(A) Comparison of the mean LDL particle size between the control and fenofibrate groups at baseline (white bar) and at follow up (gray bar)**. Bars represent the mean ± SD. (B) Correlation between mean LDL particle size and cholesterol ester transfer (CET) activity at baseline and at follow up in the control (white circle) group and fenofibrate (gray circle) group.

As shown in figure 1A, after fenofibrate treatment the mean LDL particle size increased (25.7 ± 0.6 vs. 26.1 ± 0.2 nm, *P *< 0.001). Despite the significant decrease in each LDL cholesterol level observed in the two groups, the mean LDL particle size did not change in the control group (25.8 ± 0.7 vs. 25.8 ± 0.6 nm, NS). Figure 1B shows that the increase in mean LDL particle size significantly correlated with the decrease in CET activity in all patients.

### Reduction of CET activity correlates with the decrease of triglycerides in larger HDL subclasses

**Figure 2:**
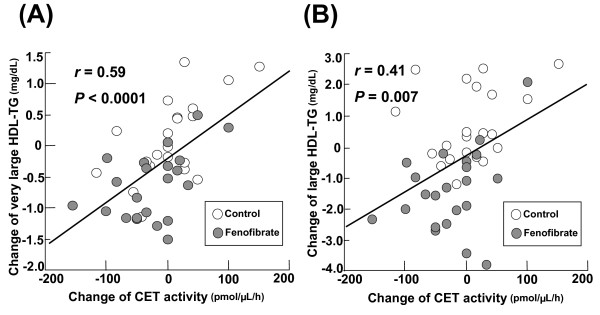
**Correlation between triglyceride levels in very large (A) and large (B) HDL subclasses and cholesterol ester transfer (CET) activity at baseline and at follow up in the control (white circle) group and fenofibrate (gray circle) group**.

Figure 2 shows the significant, positive correlation between the reduction of CET activity and the decrease of triglyceride levels in very large and large HDL subclasses. This correlation was stronger in the fenofibrate group (r = 0.48, *P *= 0.03). These results suggest that the reduction of CET activity by fenofibrate may inhibit transfer of triglycerides from apoB-containing lipoprotein to larger HDL subclasses.

### Reduction of CET activity by fenofibrate correlates with that of in-stent intimal hyperplasia

**Figure 3:**
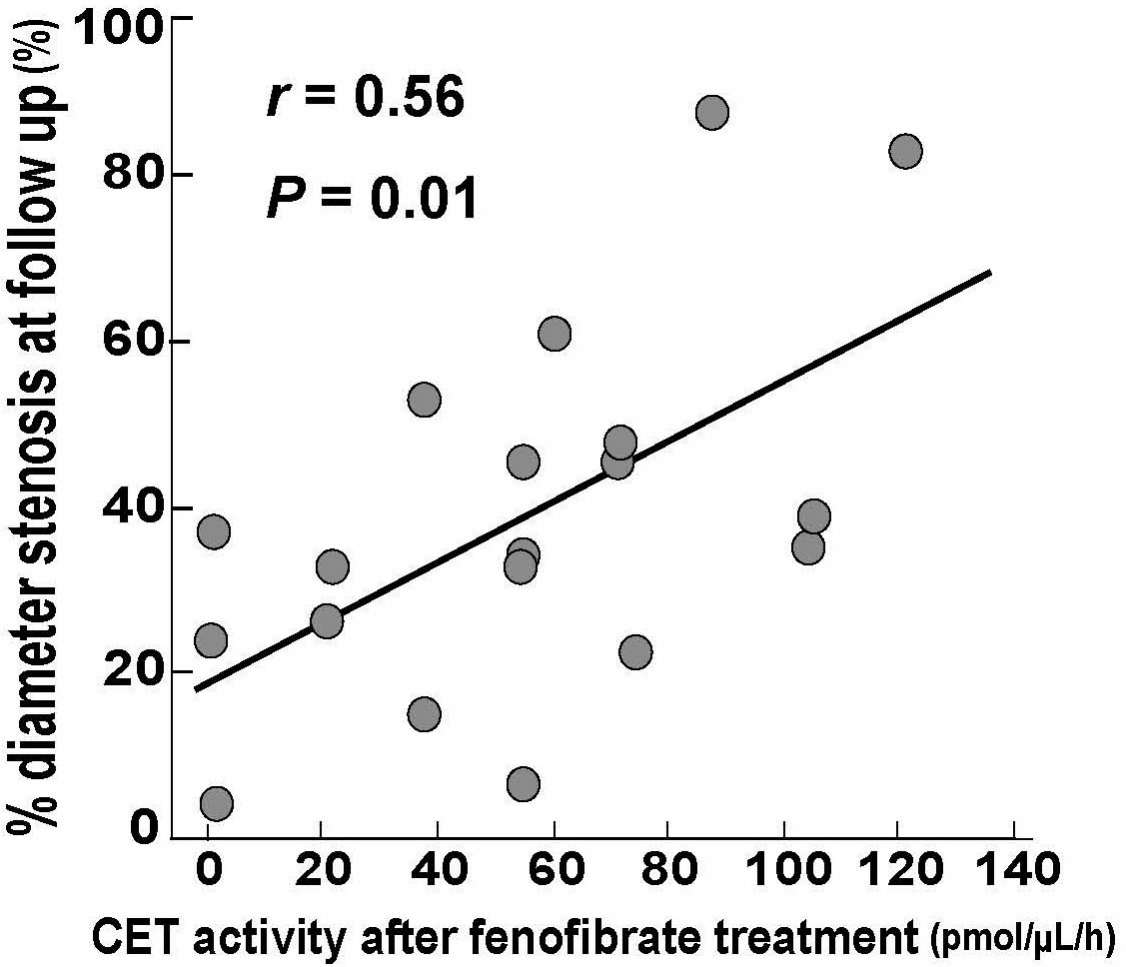
**Correlation between % diameter stenosis and cholesterol ester transfer (CET) activity at follow up**.

Table 3 shows the angiographic characteristics of patients in the two groups. There were no significant differences in angiographic findings between these groups at baseline, immediately after stenting or at follow up. There were no significant differences in restenosis parameters, such as restenosis rate, minimal luminal diameter, diameter stenosis, late lumen loss, and loss index, between the two groups. However, significant correlations were observed between CET activity and various lipid profiles in the study subjects, especially in the fenofibrate group, as mentioned above. Therefore, to investigate the effect of the reduction of CET activity on in-stent intimal hyperplasia, patients in the fenofibrate group were divided into the High CET activity (n = 9) and the Low CET activity (n = 10) groups defined as the upper and lower 50^th ^percentile of CET activity at follow up. CET activity in the High CET activity group was higher than that in the Low CET activity group (85.5 ± 22.4 vs. 30.6 ± 23.2 pmol/μL/h, *P *< 0.001). The patients in the Low CET activity group had a significantly larger minimum lumen diameter, lower % diameter stenosis, less late lumen loss, and lower loss index at follow up than patients in the High CET activity group or control group. Figure 3 indicates that after fenofibrate treatment CET activity showed a significant positive correlation with % diameter stenosis (r = 0.56, *P *= 0.01).

**Table 3:** Lesions and procedural characteristics at the time of intervention and follow-up angiography

	Control (n = 19)	Fenofibrate (n = 19)	*P*	Fenofibrate		
				
				High CET activity (n = 9)	Low CET activity (n = 10)	*P*
**Target vessel**						
LAD (%)	6 (42)	5 (24)	0.49	2 (20)	3 (27)	0.93
LCX (%)	3 (21)	6 (29)		3 (30)	3 (27)	
RCA (%)	5 (36)	10 (48)		5 (50)	5 (46)	
Complex lesion (%)	14 (74)	10 (53)	0.18	6 (67)	4 (44)	0.34
Restenosis lesion (%)	3 (16)	3 (16)	1.00	1 (11)	2 (20)	0.60
**Before stenting**						
Reference diameter (mm)	2.67 ± 0.51	2.61 ± 0.53	0.73	2.72 ± 0.61	2.60 ± 0.39	0.61
Minimal lumen diameter (mm)	0.44 ± 0.15	0.55 ± 0.20	0.10	0.59 ± 0.21	0.53 ± 0.18	0.55
Diameter stenosis (%)	82.8 ± 6.1	78.2 ± 8.6	0.09	77.4 ± 9.4	78.6 ± 8.8	0.78
Lesion length (mm)	11.8 ± 4.3	13.4 ± 6.2	0.40	12.9 ± 6.1	13.8 ± 6.8	0.78
**Procedural data**						
Maximum balloon pressure (atm)	12.9 ± 4.7	12.3 ± 4.8	0.68	12.3 ± 4.9	12.6 ± 4.8	0.80
Balloon to vessel ratio	1.13 ± 0.21	1.16 ± 0.17	0.63	1.17 ± 0.17	1.16 ± 0.18	0.88
Stent segment length (mm)	13.6 ± 3.8	13.5 ± 3.8	0.90	14.3 ± 5.0	13.2 ± 2.7	0.56
**Immediately after stenting**						
Minimal lumen diameter (mm)	2.79 ± 0.65	2.56 ± 0.50	0.26	2.56 ± 0.39	2.56 ± 0.59	1.00
Diameter stenosis (%)	9.4 ± 10.8	10.5 ± 12.8	0.79	9.7 ± 9.5	11.2 ± 15.4	0.80
**Follow-up**						
Minimal lumen diameter (mm)	1.57 ± 0.56	1.64 ± 0.60	0.73	1.35 ± 0.62	1.90 ± 0.46	0.04
Diameter stenosis (%)	44.3 ± 19.4	38.6 ± 22.0	0.44	51.9 ± 21.6	26.6 ± 14.8 *	0.01
Late lumen loss (mm)	1.22 ± 0.51	0.91 ± 0.64	0.15	1.21 ± 0.61	0.64 ± 0.56 *	0.05
Loss index Restenosis (%)	0.52 ± 0.23 5 (26)	0.44 ± 0.29 4 (21)	0.39 0.70	0.60 ± 0.28 3 (33)	0.29 ± 0.23 * 1 (10)	0.02 0.21

Data are mean ± SD. CET = cholesterol ester transfer. LAD = left anterior descending coronary artery, LCX = left circumflex coronary artery, RCA = right coronary artery. The high CET activity and low CET activity were defined as the upper and lower 50^th ^percentile of CET activity levels at follow-up, respectively. Complex lesion was defined as lesion type B2 and C according to the American College of Cardiology/American Heart Association grading system. Statistical differences of parameters between each group were analyzed by unpaired Student's *t*-test. * P < 0.05 and ** P < 0.01 vs. control group.

## Discussion

PPAR-a agonists reduce the production of atherogenic large VLDL in the liver through the stimulation of fatty acid oxidation and enhance triglyceride degradation in VLDL through the induction of lipoprotein lipase [1]. This reduction of large VLDL subsequently results in a decrease of small, dense LDL particles and an increase of HDL cholesterol [1]. In addition, PPAR-a inhibits the expression of proinflammatory genes in a ligand-dependent manner by inhibiting the action of NF-κB [19,20]. In animal models, PPAR-a also inhibits intimal hyperplasia by inhibiting vascular cell recruitment and smooth muscle cell proliferation [21,22]. Furthermore, several studies showed that PPAR-a agonists decreased CET activity in subjects with hypertriglyceridemia [12-14]. However, the association between the modulation of detailed lipoprotein profiles and CET activity by PPAR-a agonists in patients with coronary artery disease remains unclear. Therefore, we focused on the association of CET activity regulated by PPAR-a agonist, fenofibrate, with the detailed lipoprotein profiles and the development of in-stent restenosis.

Transcription of the CETP gene is induced by a high cholesterol diet in CETP transgenic mice, suggesting that fibrates may reduce CET activity through a decrease in cholesterol levels [28,29]. In our study population, however, only fenofibrate treatment significantly decreased CET activity although similar reductions of total and LDL cholesterol levels were observed in both groups. In addition, absolute changes in total and LDL cholesterol levels did not correlate with CET activity. The binding of PPAR-α to a potential peroxisome proliferator response element in the promoter region of CETP inhibited CETP gene expression [30]. These findings suggest that PPAR-α agonists directly regulated, at least in part, CET activity independent of their effects on cholesterol lowering. HPLC analysis revealed that fenofibrate significantly decreased cholesterol levels in larger VLDL subclasses and smaller LDL subclasses and increased cholesterol levels in smaller HDL subclasses. These specific changes in lipoprotein profiles, not mere cholesterol levels, also could be associated with a decrease in CET activity.

Reduction of triglycerides in apoB-containing lipoproteins by fibrates leads to an increase in LDL particle size [1]. In this study, however, the decrease in triglyceride levels in any VLDL, LDL and HDL subclass was not significantly correlated with changes in LDL size. Interestingly, the change in CET activity significantly correlated with the increase in mean LDL particle size and the decrease in cholesterol levels in smaller LDL subclasses. Changes in CET activity also correlated with triglyceride levels in larger HDL subclasses, which are the most cardioprotective HDL subclasses [23,31,32]. The reduction of CET activity results in the inhibition of the triglyceride flux from apoB containing lipoprotein to HDL. These large and triglyceride-poor HDL have a longer half-life in plasma than small and triglyceride-rich HDL [1]. Therefore, fenofibrate may inhibit removal of these cardioprotective HDL particles from the circulation through the inhibition of CET activity.

Finally, we investigated the relationship between CET activity and in-stent neointimal hyperplasia after coronary stenting. We found no significant differences in angiographical findings between the fenofibrate group and the control group at follow up. However, patients in the Low CET activity subgroup showed a significant lower % diameter stenosis and loss index than patients in the High CET activity subgroup and the control group. Interestingly, CET activity at follow up showed a significant correlation with % diameter stenosis while there were no significant correlations between lipoprotein profiles and angiographic findings. As we and another group have previously demonstrated, arterial injury induces inflammatory responses that play important roles in the occurrence of neointimal hyperplasia at the site of coronary stenting [18,26,33]. CETP inhibition increases apoA-1, paraoxonase 1 and platelet activating factor-acetylhydrolase on HDL, which have anti-inflammatory and antioxidative properties [9-11]. Furthermore, in this study, the reduction of CET activity was associated with a decrease of small LDL that is highly susceptible to oxidation [23]. We speculate that fenofibrate prevents in-stent neointimal hyperplasia through a reduction of CET activity and the subsequent induction of anti-inflammatory and antioxidative lipoprotein profiles.

This study has several limitations. First, the sample size was small, especially in the High and Low CET activity groups. Therefore, we could not perform multivariate analysis to exclude the interaction among lipoprotein subclasses, CET activity and angiographic findings. We believe that the present study is one of the pioneering and valuable reports promoting further investigations. Second, we could not exclude the effects of changes in triglycerides and HDL cholesterol induced by fenofibrate from those on CET activity. However, there were no significant correlations between changes in CET activity and changes in total triglycerides or HDL cholesterol. Third, we did not measure CETP mass in this population. Therefore, whether the reduction of CET activity depends on the production of CETP remains unclear. Fourth, all patients were performed bare metal but not drug-eluting stenting. However, as mentioned above, in-stent restenosis still remains a clinical problem even in drug-eluting stent era [16]. In addition, we did not perform intravascular ultrasound for quantitative assessment in this study. The assessment using intravascular ultrasound is needed in the next step.

## Conclusions

In conclusion, fenofibrate decreased CET activity independent of its lowering effect on total and LDL cholesterol in patients with coronary artery disease. That reduction of CET activity significantly correlated with any increase in LDL particle size and a decrease in triglycerides in larger HDL subclasses, besides a decrease in in-stent intimal hyperplasia. Inhibition of CET activity by fenofibrate might be a potent strategy for preventing in-stent intimal hyperplasia after coronary stenting.

## Competing interests

The authors declare that they have no competing interests.

## Authors' contributions

TM participated in planning of the study, experimental work, analysis and prepared the manuscript. KazS contributed in designing of the study, data analysis and publication of results. AK, KatS, and TK participated in lipid analysis and CETA measurement. HM participated in planning of the study and discussion of results. KT, TK, MH, SO, HS, and TK involved in coronary catheterization. KM and HD contributed in planning of the experiment and discussion of results, and supervised the study. All authors read and approved the manuscript.
